# A novel vaccine construct against Zika virus fever: insights from epitope-based vaccine discovery through molecular modeling and immunoinformatics approaches

**DOI:** 10.3389/fimmu.2024.1426496

**Published:** 2024-07-01

**Authors:** Metab Alharbi, Abdulrahman Alshammari, Jawza F. Alsabhan, Sami I. Alzarea, Talal Alshammari, Fawaz Alasmari, Abdullah F. Alasmari

**Affiliations:** ^1^ Department of Pharmacology and Toxicology, College of Pharmacy, King Saud University, Riyadh, Saudi Arabia; ^2^ Department of Clinical Pharmacy, College of Pharmacy, King Saud University, Riyadh, Saudi Arabia; ^3^ Department of Pharmacology, College of Pharmacy, Jouf University, Sakaka, Aljouf, Saudi Arabia

**Keywords:** epitopes base vaccine construct, molecular docking, molecular dynamic simulation, Zika virus, binding free energies

## Abstract

The Zika virus (ZIKV) is an emerging virus associated with the *Flaviviridae* family that mainly causes infection in pregnant women and leads to several abnormalities during pregnancy. This virus has unique properties that may lead to pathological diseases. As the virus has the ability to evade immune response, a crucial effort is required to deal with ZIKV. Vaccines are a safe means to control different pathogenic infectious diseases. In the current research, a multi-epitope-based vaccination against ZIKV is being designed using *in silico* methods. For the epitope prediction and prioritization phase, ZIKV polyprotein (YP_002790881.1) and flavivirus polyprotein (>YP_009428568.1) were targeted. The predicted B-cell epitopes were used for MHC-I and MHC-II epitope prediction. Afterward, several immunoinformatics filters were applied and nine (REDLWCGSL, MQDLWLLRR, YKKSGITEV, TYTDRRWCF, RDAFPDSNS, KPSLGLINR, ELIGRARVS, AITQGKREE, and EARRSRRAV) epitopes were found to be probably antigenic in nature, non-allergenic, non-toxic, and water soluble without any toxins. Selected epitopes were joined using a particular GPGPG linker to create the base vaccination for epitopes, and an extra EAAAK linker was used to link the adjuvant. A total of 312 amino acids with a molecular weight (MW) of 31.62762 and an instability value of 34.06 were computed in the physicochemical characteristic analysis, indicating that the vaccine design is stable. The molecular docking analysis predicted a binding energy of −329.46 (kcal/mol) for TLR-3 and −358.54 (kcal/mol) for TLR-2. Moreover, the molecular dynamics simulation analysis predicted that the vaccine and receptor molecules have stable binding interactions in a dynamic environment. The C-immune simulation analysis predicted that the vaccine has the ability to generate both humoral and cellular immune responses. Based on the design, the vaccine construct has the best efficacy to evoke immune response in theory, but experimental analysis is required to validate the *in silico* base approach and ensure its safety.

## 1 Introduction

Viral infections recently emerged as a global health problem. Over the last few years, the understanding of different viral infections has changed with the discovery of new virulence strategies with associated hosts and modes of infection (1). Among viral infections, the propagation of the Zika viral infection has also become an alarming worldwide health issue. Zika virus infection is rapidly spreading to different countries due to several approaches, but transmission mainly occurs through traveling (2). The Zika virus initially appears to cause moderate to severe infection; the long-term infection is more overwhelming to the next generation and affects the fetus as well. The Zika virus is a developing viral infection from *Flaviviridae*, and it is mainly transmitted to humans by mosquitoes. The infection mainly occurs in pregnant women and can affect the fetus as well and may lead to microcephaly in new newborns (3). The Zika virus has a unique capability to use the host system to enhance the replication process in a tissue-specific manner, leading to pathological events. Recent research has proposed that the Zika virus has evasion and tropism properties, wherein the virus not only evades immune cells and causes severe illness, but also enhances entry to new host cells (4).

In addition, the Zika virus has established a mechanism to evade immune cells, thus allowing the establishment of viral persistence, and it is increasing the pathogenicity. As the virus has become a global health problem and is efficiently transmitted from country to country, there is a need to eradicate Zika virus through vaccination (5). The vaccination process is a cornerstone of global health protocol, and it is demonstrated to be highly effective in dealing with microbial infections (6).

Considering the life threatening Zika virus complications, developing vaccine against the virus could be the best option to prevent the viral infections. As several vaccines against the Zika virus are under clinical phase, a multi-epitope vaccine construct could be an attractive option for future vaccine development against the virus (7). Epitope vaccine development against the Zika virus is less expensive, less time-consuming, and less labor-intensive; thus, it is an effective and safe approach to designing a vaccine against the Zika virus (8).

Pasture vaccinology-based vaccines contributed much to vaccine development, but in most cases, such an approach for vaccine development has failed due to several reasons like culturing of several pathogenic viruses and the spread of their respective infections (8). Furthermore, it is also clear that there is a major challenge in vaccine development such as proper vaccine candidate identification in pathogenic microorganisms (9). Furthermore, immunologists should take the lead in developing multi-epitope-based vaccine constructs because of the difficulties associated with using vaccines to prevent disease outbreaks and safeguard the elderly population, as well as the accessibility of computer-aided vaccine designing (10).

Multi-epitopes can be considered a promising approach against tumors and viral infections (11). Epitope vaccines consist of MHC-restricted epitopes that can be recognized by the immune cells of multiple clones from various B cells and B-cell-derived T-cell epitopes that can induce proper immune responses against targeted pathogens and provide long-lasting immunity (12). Multi-epitope vaccine constructs can reduce harmful effects, as during the development of epitope-based vaccine constructs, all the unwanted allergic components are mainly removed during immunoinformatics approaches (13). Multi-epitope-based vaccine constructs have several advantages, hence becoming a powerful tool for prophylactic and therapeutic agents against tumor, bacterial, and viral infection (14). Thus, current work has applied several bioinformatics, immunoinformatics, and several biophysical pipelines to develop a multi-epitope-based vaccine construct against the Zika virus in tackling this health-related issue.

## 2 Research methodology

**Figure 1:**
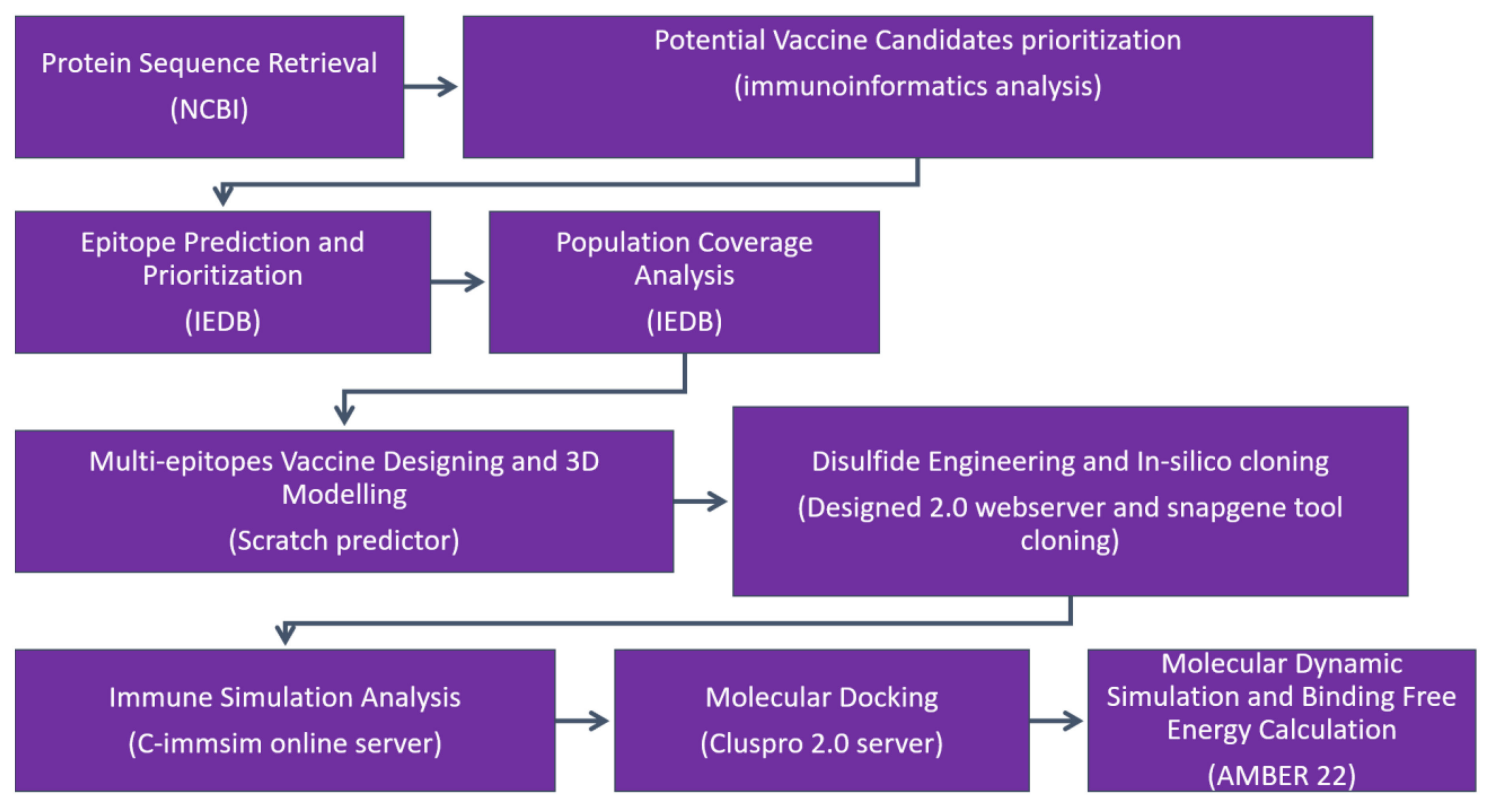
The research methodology flow is as follows: (1) protein retrieval, where the immunogenic proteins of the targeted virus is retrieved; (2) potential vaccine candidate prioritization; (3) epitope prediction and prioritization analysis; (4) population coverage analysis; (5) vaccine design and processing phase; (6) disulfide engineering and *in silico* analysis; (7) immune simulation analysis of selected epitopes; (8) molecular docking; (9) molecular dynamic simulation analysis; and (10) binding free energy calculation.

The research methodology flow diagram is presented in **Figure 1**.

### 2.1 Protein retrieval

In the protein target selection phase, GCA_0008828151, GCA_0023662851, GCA_0047871951, GCA_0047877351, and GCA_0047881551 of the Zika virus were selected and sequences of the selected proteins were obtained from the National Center for Biotechnology Information (NCBI) (15). In the physicochemical property analysis, molecular weight, theoretical pI, instability index, aliphatic index, and grand average of hydropathy (GRAVY) were checked and only physicochemically stable proteins were selected for immunoinformatics analysis (16). In immunoinformatics analysis, antigenicity, allergenicity, water solubility, and homology were predicted using the vaxijen 2.0 tool, the peptide solubility calculator, and BLASTp online web tools. In immunoinformatics analysis, only probable antigenic, non-allergenic, water-soluble, and human non-similar proteins were selected for the epitope prediction phase (14).

### 2.2 Potential vaccine candidate prioritization

In the potential vaccine candidate selection phase, we performed several immunoinformatics analysis like antigenicity and allergenicity using vaxijen (17) Allertop 2.0 (18). To identify soluble protein sequences, we analyzed the protein sequences using the Innovogen server and conducted an adhesion probability check using the vaxijen 2.0 tool (12). Lastly, we ran a BLASTp search against human and *Lactobacillus* species for these protein sequences (19).

### 2.3 Epitope prediction and prioritization

We utilized the Immune Epitope Analysis Database (IEDB) (20) to perform epitope mapping on the protein sequences that met our criteria as potential candidates and predicted epitopes (21). The predicted B-cell epitopes were then used for T-cell epitope prediction (MHC-I and MHC-II) (6). We chose a whole set of reference alleles for T-cell epitope mapping, using both MHC I and MHC II binding epitopes (22). Using MHCpred, the binding affinity of these T-cell epitopes produced from B cells was examined; epitopes with an IC_50_ value of less than 100 were deemed to be good binders. The antigenic, non-allergenic, non-toxic, and soluble epitopes that were shortlisted were utilized to develop the multiepitope vaccine construct (7).

### 2.4 Population coverage analysis

We conducted a population coverage analysis using the “Population Coverage” tool from IEDB (23). This analysis aimed to determine the coverage of the shortlisted epitopes for alleles that represent the global population (24). The goal was to design a multiepitope vaccine that can target a large portion of the world population (25).

### 2.5 Multi-epitope vaccine designing and 3D modeling

To design the vaccine construct, we connected the shortlisted epitopes using GPGPG linkers (8). Additionally, we used the EAAAK linker to attach to the Cholera toxin B subunit (CTBS) adjuvant, which enhances the vaccine’s activity. The Cholera toxin B subunit is a powerful mucosal adjuvant for the generation of mucosal antibody responses and specific immunity, and it has been experimentally validated. Furthermore, the CTB subunit was shown to bind with a high affinity specifically to the cellular receptor and can trigger cellular immune responses (26). Different linkers were used to separate epitopes from each other to avoid folding of epitopes during structure modeling. The physicochemical characteristics and “antigenicity, allergenicity, adhesion probability”, as well as the secondary structure parameters of the construct were analyzed (27). The final vaccine construct’s molecular weight, hydrophilicity, and theoretical and instability index were obtained using the Protparam tool from Expasy (25). To predict the 3D structure of the vaccine design, we utilized the Scratch predictor (28). The vaccine construct, designed with epitopes, was submitted to the Scratch predictor for 3D structure prediction. The presence of loops in the vaccine model was analyzed, followed by loop modeling and structure refinement using Galaxy Loop and Galaxy Refine of Galaxy Web (29).

### 2.6 Disulfide engineering and *in silico* cloning

Using the Disulfide by Design 2 online server, disulfide engineering was carried out to improve the stability of the vaccine model. This involved mutating potentially unstable residues into cysteine residues, facilitating the formation of disulfide bonds and increasing structural stability (30). The Java Codon Adaptation Tool (JCat) was used to optimize the vaccine construct’s codons (26). The vaccine sequence was reverse-translated, and parameters such as codon adaptation index (CAI) and GC content were obtained. The resulting optimized sequence was then *in silico* cloned using Snapgene (31), and expressed in the pET-28a (+) expression vector (32).

### 2.7 Immune simulation analysis

The analysis of immunogenicity and immune-inducing potential of vaccine constructs can be accomplished through computational immune simulation. This approach utilizes the C-ImmSim server (33), which incorporates machine learning techniques to predict the interaction between immune epitopes (34).

### 2.8 Molecular docking

To investigate the interconnection between the vaccine model and receptor, a molecular docking analysis was conducted (35). TLR receptors play a crucial role in triggering cytokine production, which, in turn, activates the innate immune response (25). The ClusPro 2.0 (36) online tool was utilized for the analysis of docking (37). The intermolecular interactions between the vaccine and receptor were predicted using different visualization software applications (13).

### 2.9 Molecular dynamics simulation and binding free energy calculation

In this research, we utilized the AMBER18 software for molecular dynamics (MD) simulation of the vaccine and TLR complex (38). The “ff14SB” force field was deployed to set the parameters for both the TLR and vaccine (27). The system was simulated in an aqueous solution using a TIP3P water box with a padding distance of 12 Å. Na+ ions were added for system neutralization, and steps were taken to optimize the system (39). The system was heated to 300K for 20 ps and then gradually stabilized. The production run of 50 ns was carried out using the NPT ensemble. AMBER CPPTRAJ was used for trajectory analysis, including hydrogen bonding analysis between the vaccine and TLR4. MM-PB/GBSA studies were also conducted (25).

## 3 Results

### 3.1 Protein retrieval

In protein retrieval, GCA_0008828151, GCA_0023662851, and GCA_0047871951 proteins were retrieved and analyzed for physicochemical properties analysis, in which the amount of amino acids, instability index, theoretical pI, molecular weight, GRAVY, and aliphatic index were examined as shown in **Table 1**. In physicochemical properties analysis, an instability index of 46.7 was computed for GCA_0047871951, which is greater than the cutoff value of 40; hence, the protein was considered unstable and discarded from further analysis, while the other two proteins, GCA_0008828151 and GCA_0023662851, were utilized for further immunoinformatics analysis. In immunoinformatics, antigenicity, allergenicity, and water solubility were assessed and both proteins were found to be probably antigenic with 0.5191 and 0.5201 predicted values. Moreover, the proteins were also predicted to be a probable non-allergen and to have good water solubility. **Table 2** depicts overall findings of immunoinformatics analysis.

**Table 1:** Physicochemical characteristics of targeted proteins of the Zika virus.

Targeted proteins of the Zika virus	Physicochemical property analysis					
	Number of amino acids	Molecular weight	Theoretical pI	Instability index	Aliphatic index	GRAVY
GCA_0008828151	3,419	37.87	8.64	37.33	86.93	−0.14
GCA_0023662851	3,423	37.90	8.63	36.86	86.52	−0.14
GCA_0047871951	7,770	62.71	4.64	46.7	27.01	0.76

**Table 2:** Immunoinformatics analysis profiling of selected proteins.

Selected proteins for epitope prediction	Immunoinformatics analysis		
	Antigenicity	Allergenicity	Water solubility
GCA_0008828151	0.51 (Antigenic in nature)	Probable non-allergenic	Good water solubility
GCA_0023662851	0.52 (Antigenic in nature)		

### 3.2 Epitope prediction and prioritization

**Figure 2:**
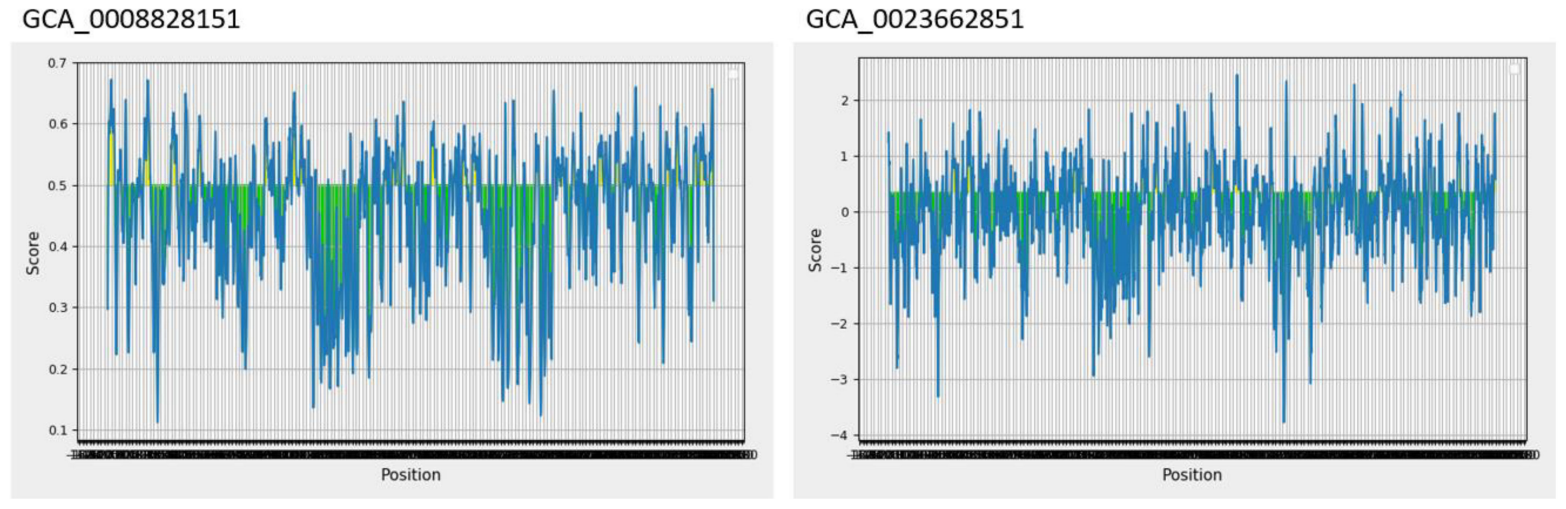
Predicted B-cell epitopes; the yellow color represents B-cell peptides generated by the Immune Epitope Database (IEDB).

Epitopes are antigenic determinant short sequences of amino acids that are mainly exposed on the surface of molecules and can easily evoke the right immune responses in the host body against specific antigens. In the epitope prediction phase, probable non-allergenic, antigenic, and water-soluble GCA_0008828151 and GCA_0023662851 proteins were used for B-cell epitope prediction analysis as the predicted B-cell epitopes are presented in **Table 3**, while in **Figure 2**, the yellow peaks in the graph represent B-cell-predicted epitopes. The B-cell epitopes were further used for T-cell epitope prediction; in T-cell epitope prediction, both MHC-I and MHC-II epitopes were predicted and are shown in **Supplementary Table 1 (S1)**.

**Table 3:** B-cell epitopes with various lengths.

Selected proteins	B-cell epitopes
GCA_0008828151	KEEIRRIRIVNMLKRGVARVNPLGGLKR LPAGLLLGH
	IKPSLGLINRWGSVGKKE
	CHHKKGEARRSRRAVTLPSHSTRKLQTRSQT WLESREYTK
	SDMASDSRCPTQGEAYLDKQSDTQYVCKRTL VDRGWGN
	FHDIPLPWHAGADTGTPHWNNKE
	VKNPMWRGPQRLPVPVNELPHGWKAWGKS
	AGPLSHHNTREGYRTQVKGPWHSEEL
	ETCGTRGPSLRSTTASGRVIEEWCCRECTMP
	ALRSGEGRLDPYWGDVKQDLVSYCGPWKL DAAWDG
	VVIKNGSYVSAITQGKREEETPVEC
	YTDRRWCFDGTTNNTIMEDSVPAEVWT KYGEKRV
	NPVVDGIVVTDIDTMTIDPQVE
	PRRPVKYEEDVNLGSGTRAVASCAEAPNMKIIGR
	TTPYGQQRVFKEKVDTRVPDPQEGTR
	VLEMQDLWLLRKPEKVTRWLQSNGWDR
	GKVRKDTQEWKPSTGWSNWE
	ELIGRARVSPGAGWSIRE
	DWVPTGRTTWSIHGKGEWMTTE
	NDHMEDKTPVTKWTDIPYLGKREDLWCG SLIGHRPRTTWA
	GDEEKYMDYLSTQVRYLGEEGSTP
GCA_0023662851	PKKKSGGFRIVNMLKRGVARVSPFGGL KRLPAGLLLGH
	IKPSLGLINRWGSVGKKE
	CHHKKGEARRSRRAVTLPSHSTRKLQTRS QTWLESREYTK
	SDMASDSRCPTQGEAYLDKQSDTQYVC KRTLVDRGWG
	SQHSGMIVNDTGHETDEN
	VKNPMWRGPQRLPVPVNELPHGWKAWGK
	TCGTRGPSLRSTTASGRVIEEWCCRECTMP
	TRDAFPDSNSPIMDTEVEVPERAWSSGFD WVTDHS
	LQDGLIASLYRPEADKVAAIEGEFKLRTE
	TYTDRRWCFDGTTNNTIMEDSVPAEVWTR HGEK
	NPVVDGIVVTDIDTMTIDPQVE
	MSALEFYSYKKSGITEVCREE
	RRALKDGVATGGHAVSRGSA
	RRPVKYEEDVNLGSGTRAVVSCAE APNMKIIGN
	TTPYGQQRVFKEKVDTRVPDPQEGT
	RFWALVDKEREHHLRGE
	MMGKREKKQGEFGKAK
	VLEMQDLWLLRRSEKVTNWLQSNGWDRL
	GKVRKDTQEWKPSTGWDNWEEVPFCSHHF
	IGRARVSPGAGWSIRE
	DWVPTGRTTWSIHGKGEWMTTE
	NDHMEDKTPVTKWTDIPYLGKREDLWCGS LIGHRPRTTW
	GDEEKYMDYLSTQVRYLGEEGSTP

Additionally, the predicted MHC-I and MHC-II epitopes were further used for MHC-Pred Analysis. The predicted epitopes were assessed for binding affinity with HLA-DRB1*0101 in the said analysis, and only good HLA-DRB1*0101 binder epitopes were shortlisted, while poor HLA-DRB1*0101 binder epitopes were discarded. Moreover, the selected epitopes were additionally used and examined for antigenicity, allergenicity, toxicity, and water solubility analysis, and in the said analysis, probable non-allergenic, antigenic, non-toxic, and water-soluble epitopes were used for multi-epitope vaccine constructs; the selected epitopes are shown in **Table 4**.

**Table 4:** Selected epitopes for the multi-epitope vaccine construct.

S. no.	MHC-Pred analysis	Predicted IC_50_ value (nM)	Allergenicity	Antigenicity	Cutoff value	Water solubility	Toxicity
1	EARRSRRAV	28.91	Probable non-allergenic	Probable good antigenic	0.91	Good water solubility	Non-toxic
2	AITQGKREE	90.78			1.69		
3	ELIGRARVS	6.03			0.49		
4	KPSLGLINR	74.64			1.06		
5	RDAFPDSNS	54.2			0.40		
6	TYTDRRWCF	3.74			2.24		
7	YKKSGITEV	40.74			0.67		
8	MQDLWLLRR	3.23			0.56		
9	REDLWCGSL	44.57			1.62		

### 3.3 Worldwide coverage of vaccine candidate

**Figure 3:**
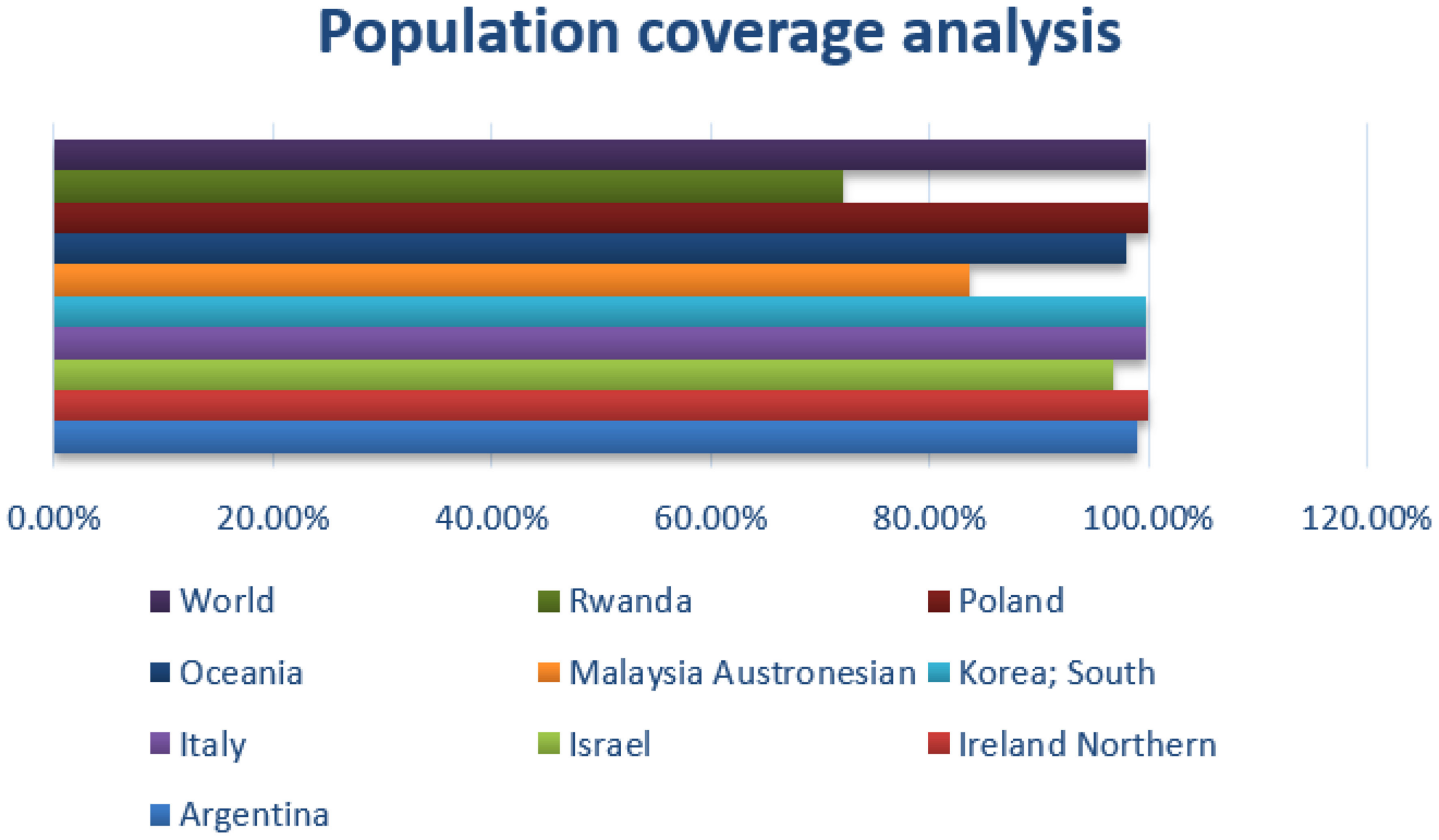
Combined MHC-I and MHC-II; population coverage analysis of the selected epitopes; query by area, country, and ethnicity of population.

In the population coverage analysis, the immune efficacy of the selected epitopes was analyzed, and the analysis of population coverage predicted that the vaccine candidates can evoke proper immune responses. **Figure 3** presents population coverage analysis results.

### 3.4 Vaccine construction and processing phase

**Figure 4:**
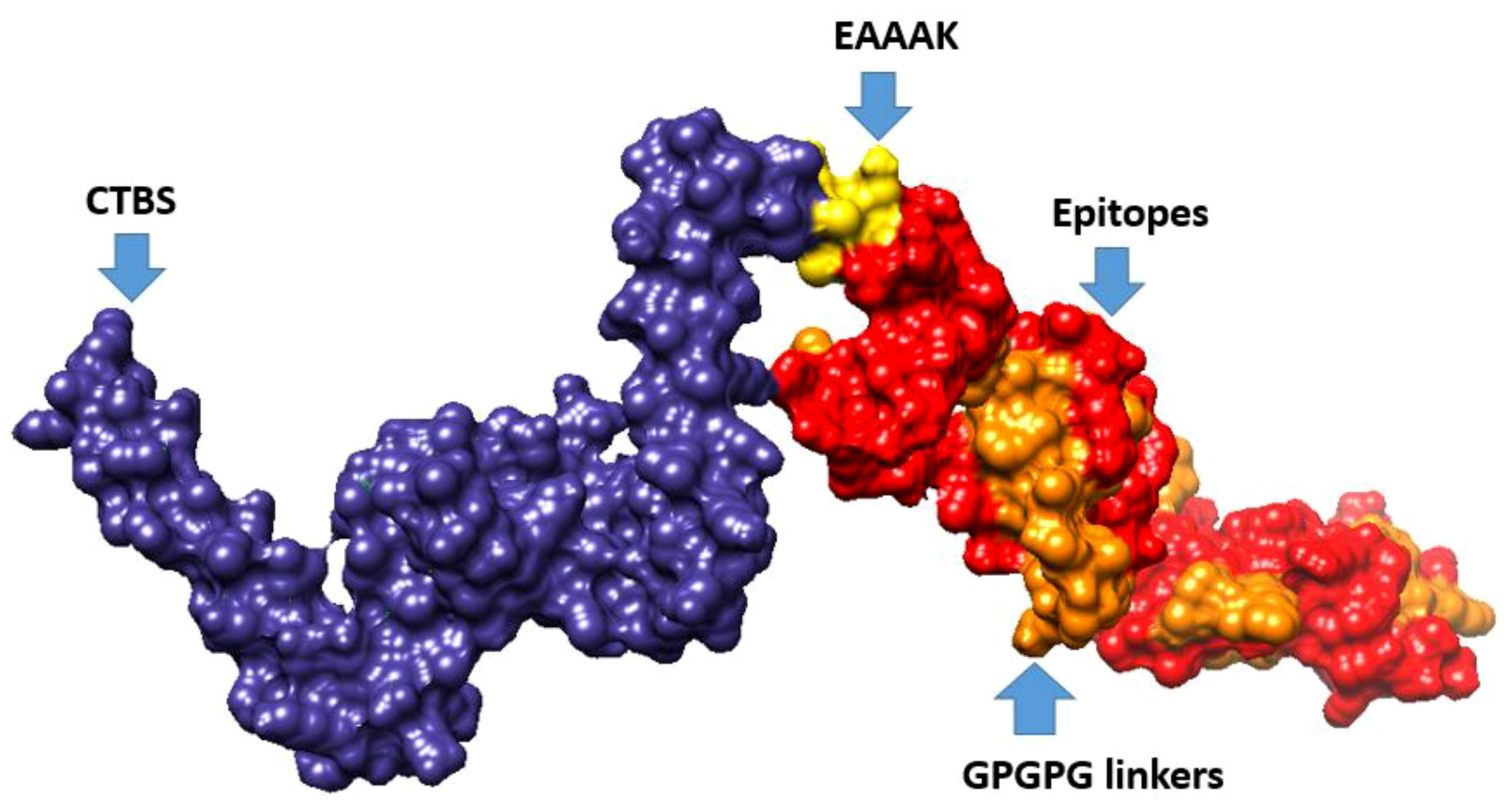
3D representation of multi-epitope vaccine candidates, composed of “Cholera toxin B subunit adjuvant (CTBS)”, EAAAK, epitopes, and GPGPG linkers.

Multi-epitope vaccine constructs are composed of a series of probable antigenic epitopes and can easily evoke appropriate immune responses in the host. Multi-epitope vaccine constructs were designed using different linker selected epitopes and adjuvants. EAAAK linkers were used for linking vaccine constructs with adjuvant, while the GPGPG linker was used for joining selected epitopes. **Figure 4** shows the 3D structure of the vaccine design. The GalaxyWEB internet webserver was utilized to further enhance the vaccine design structure, as indicated by the projected values presented in **Table 5**.

**Table 5:** GalaxyWEB refinement of vaccine construct models.

Model	GDT-HA	RMSD	MolProbity	Clash score	Poor rotamers	Rama favored
Initial	1	0	1.93	14.6	0.9	96.1
Model 1	0.98	0.31	1.75	11.9	0.5	97.1
Model 2	0.98	0.32	1.77	11.5	0.9	96.8
Model 3	0.97	0.32	1.68	10.2	0.9	97.1
Model 4	0.99	0.3	1.65	10.6	0.9	97.4
Model 5	0.9	0.31	1.73	11.5	0.5	97.1

### 3.5 Analysis of disulfide engineering

**Figure 5:**
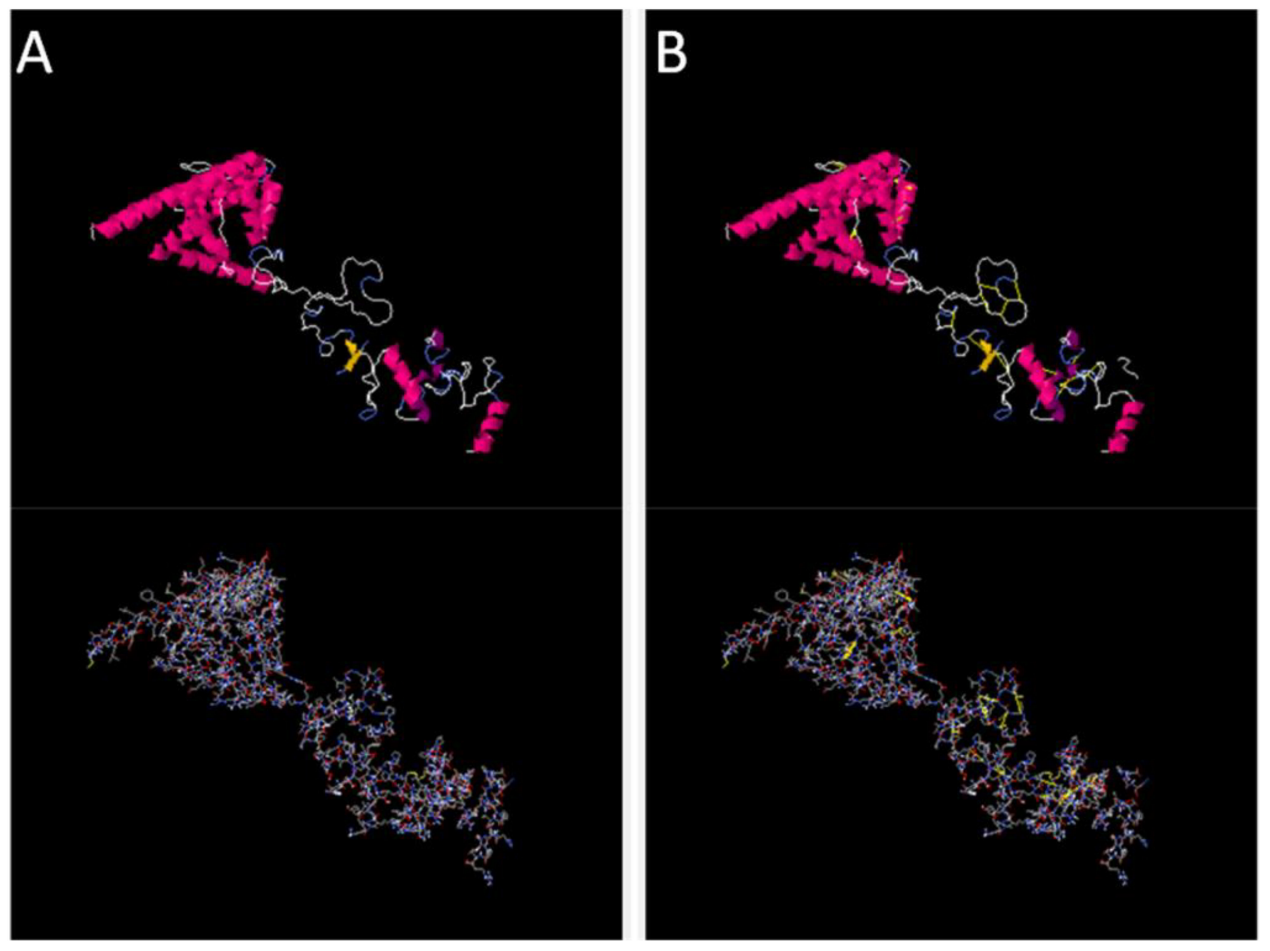
Original and mutated structure of the vaccine construct. **(A)** represents the wild structure and **(B)** represents the mutated structure of the vaccine construct; the yellow sticks represent amino acids replaced by cysteine amino acid residues.

Disulfide engineering was performed using the designed 2.0 online web server. The server predicted several amino acid residues to be replaced with cysteine amino acid residues in order to maintain the stability of the designed vaccine construct. In **Figure 5**, cartoon and wireframe structures of the wild and mutant vaccine construct are presented; moreover, the amino acid residues Chi 3 energy and sum beta factors are mentioned in **Supplementary Table S2.**

Furthermore, *in silico* cloning was performed using the snapgene tool; the vaccine construct sequence was first converted into the DNA sequence

“ATGGCTAAACTGTCTACCGACGAACTGCTGGACGCTTTCAAAGAAATGAC

CCTGCTGGAACTGTCTGACTTCGTTAAAAAATTCGAAGAAACCTTCGAAG

TTACCGCTGCTGCTCCGGTTGCTGTTGCTGCTGCTGGTGCTGCTCCGGCT

GGTGCTGCTGTTGAAGCTGCTGAAGAACAGTCTGAATTCGACGTTATCCT

GGAAGCTGCTGGTGACAAAAAAATCGGTGTTATCAAAGTTGTTCGTGAAA

TCGTTTCTGGTCTGGGTCTGAAAGAAGCTAAAGACCTGGTTGACGGTGCT

CCGAAACCGCTGCTGGAAAAAGTTGCTAAAGAAGCTGCTGACGAAGCTAA

AGCTAAACTGGAAGCTGCTGGTGCTACCGTTACCGTTAAAGAAGCTGCTG

CTAAAGAAGCTCGTCGTTCTCGTCGTGCTGTTGGTCCGGGTCCGGGTGCT

ATCACCCAGGGTAAACGTGAAGAAGGTCCGGGTCCGGGTGAACTGATCGG

TCGTGCTCGTGTTTCTGGTCCGGGTCCGGGTAAACCGTCTCTGGGTCTGA

TCAACCGTGGTCCGGGTCCGGGGCGTGACGCGTTCCCGGACTCTAATTCT

GGTCCGGGTCCGGGTACCTACACCGACCGTCGTTGGTGCTTCGGTCCGGG

TCCGGGTTACAAAAAATCTGGTATCACCGAAGTTGGTCCGGGTCCGGGTA

TGCAGGACCTGTGGCTGCTGCGTCGTGGTCCGGGTCCGGGTCGTGAAGAC

CTGTGGTGCGGTTCTCTGGGTCCGGGTCCGGGTTCTGACATGGCTTCTGA

CTCTCGTTGCGGTCCGGGTCCGGGTGGTACCCGTGGTCCGTCTCTGCGTT

CTGGTCCGGGTCCAGGTCGTGACGCGTTCCCGGACTCTAATTCTGGTCCG

**Figure 6:**
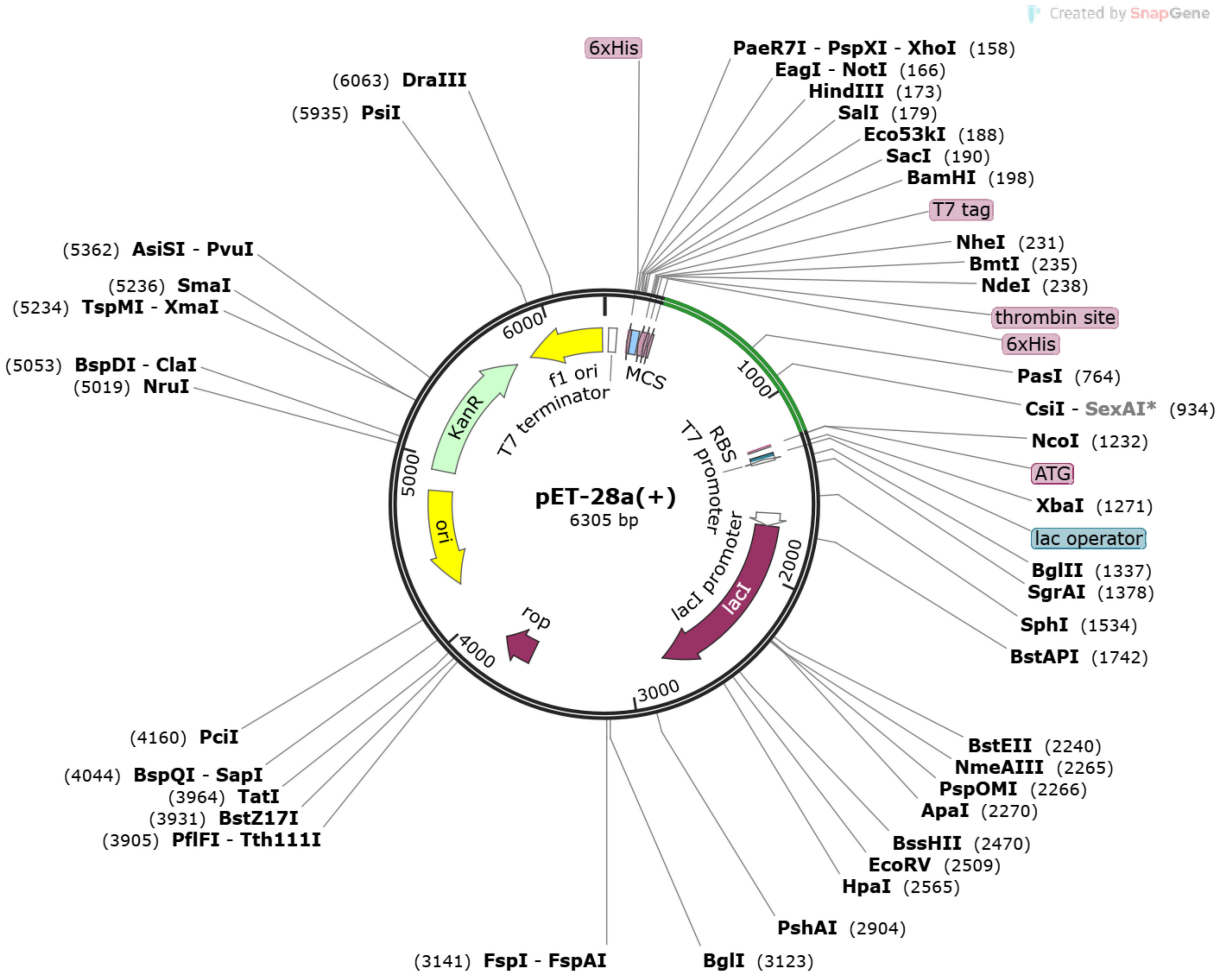
Pet-28 (+) cloned vector. The light green line represents inserted sequences after the 6×HIS site.

GGTCCGGGTGTTACCCACGCTTCTGCTGCTCAGCGT” using codon usage adapted to *Escherichia coli* (strain K12) as presented in **Supplementary Figure S1**. Moreover, the cloned sequence in the pet vector map after adaptation is presented in **Figure 6**.

### 3.6 Analysis of molecular docking

**Figure 7:**
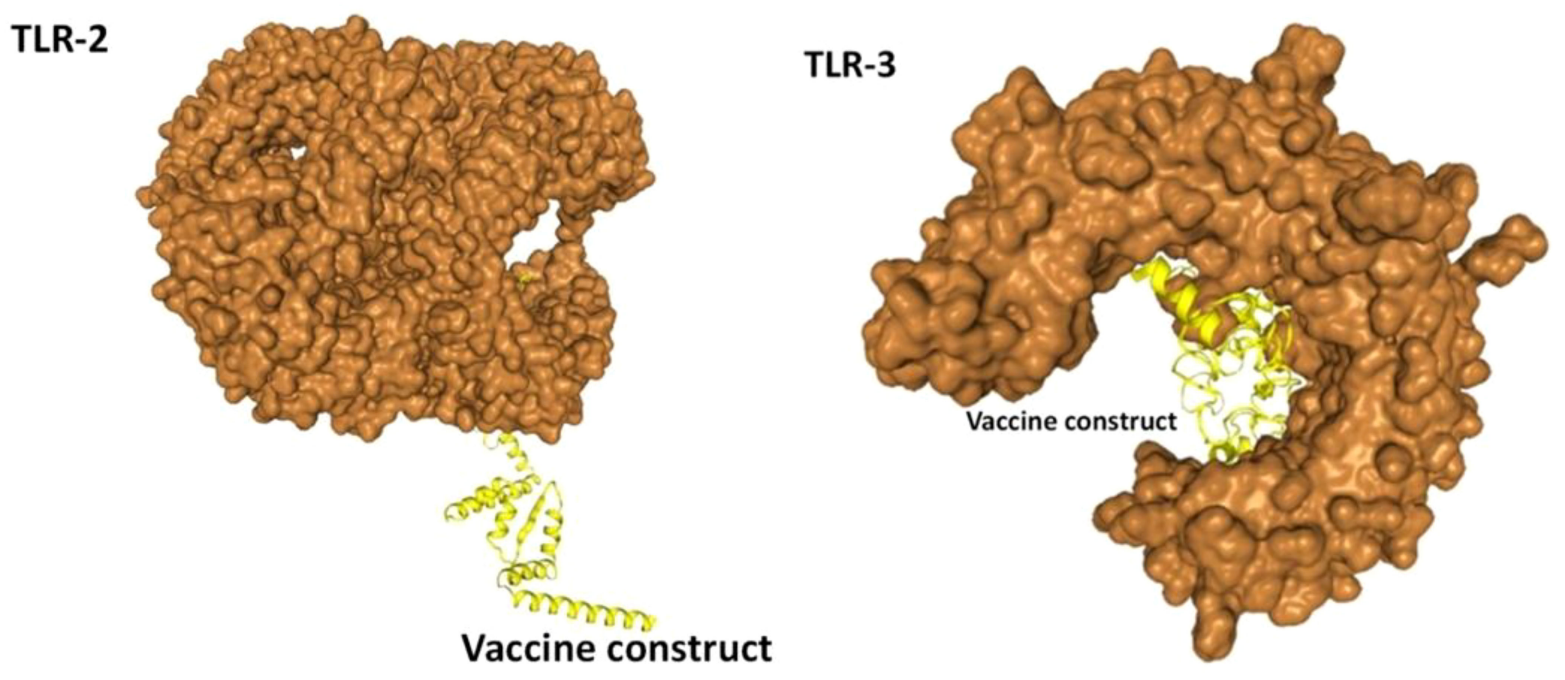
Vaccine with docked receptors. The TLR-2 and TLR-3 are shown in surface brown while the yellow carton shape represents the multi-epitope vaccine construct.

In the analysis of molecular docking, the vaccine construct was analyzed for binding ability with immune cell receptors (TLR-2 and TLR-3). Docking analysis generated top dock complexes based on docking score, confidence score, and ligand rmsd (Å). The vaccine and TLR-2 docking results are mentioned in **Table 6**, while in **Table 7**, docking results of vaccine and TLR3 are tabulated. The intermolecular docked confirmation of the vaccine construct with TLR-2 and TLR-3 is presented in **Figure 7**.

**Table 6:** Vaccine and TLR-2 docking analysis.

Vaccine and TLR-2										
Rank	1	2	3	4	5	6	7	8	9	10
Docking score	−329.46	−303.98	−288.8	−275.66	−267.96	−259.44	−257.8	−254.07	−246.15	−243.39
Confidence score	0.9731	0.956	0.9414	0.9251	0.9137	0.8992	0.8962	0.8891	0.8725	0.8662
Ligand rmsd (Å)	197.54	210.59	206.77	211	201.58	233.55	220.13	229.4	188.62	189.21
Interface residues	model_1	model_2	model_3	model_4	model_5	model_6	model_7	model_8	model_9	model_10

**Table 7:** Vaccine and TLR-2 docking analysis.

Vaccine and TLR-3										
Rank	1	2	3	4	5	6	7	8	9	10
Docking score	−358.54	−312.92	−310.43	−309.66	−307.85	−307.32	−299.46	−296.35	−294.56	−293.79
Confidence score	0.9848	0.963	0.9612	0.9606	0.9592	0.9588	0.9521	0.9492	0.9474	0.9466
Ligand rmsd (Å)	153.07	156.44	136.22	151.76	162.38	122.55	138.75	97.59	127.38	116.45
Interface residues	model_1	model_2	model_3	model_4	model_5	model_6	model_7	model_8	model_9	model_10

### 3.7 Root mean square deviation analysis

**Figure 8:**
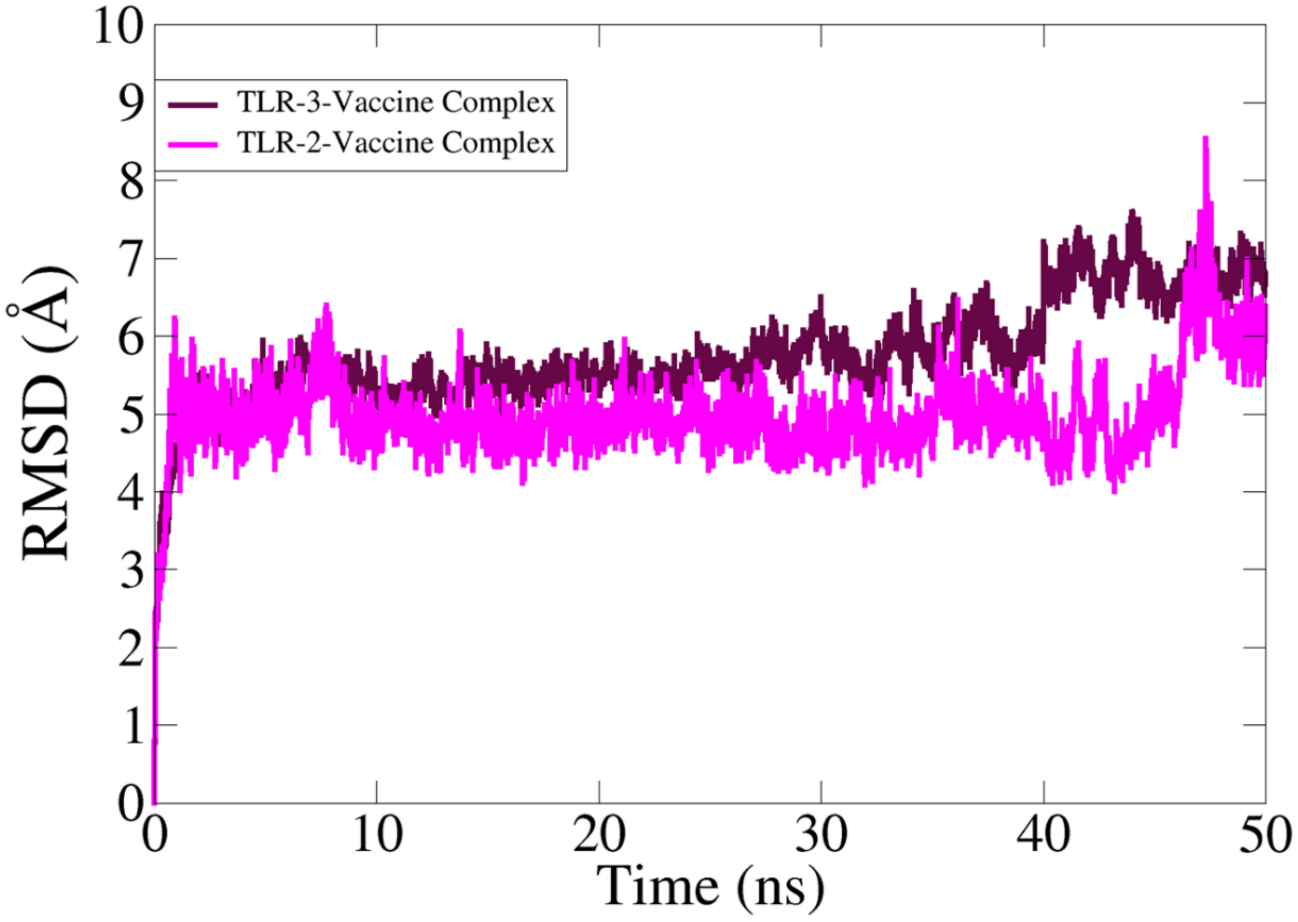
Simulation trajectory analysis and root mean square deviation analysis.

In MD simulation analysis, the dynamic behavior of the vaccine with the targeted receptors is determined. In MD simulation, root mean square deviation (RMSD) analysis was evaluated with 50 ns. The RMSD of TLR-2 showed more stability compared to TLR-3 as the RMSD of TLR-3 deviated more with respect to simulation time. The TLR-2 graph showed maximum instability, but the graph becomes stable at the end of simulation time as compared to TLR-3. The simulation trajectory showed that the vaccine molecule and TLR-2 and TLR-3 have the best binding strength, which can evoke proper host immunity against vaccine candidates. The RMSD graphs of TLR-2 and TLR-3 are presented in **Figure 8**.

### 3.8 MMGB/PBSA analysis

The binding free energy of the vaccine construct and receptor was calculated using MMGBSA and MMPBSA in the AMBER 21 package. In the MMGBSA analysis for vaccine and TLR-2, van der Waals energy, coulombic energy, total gas phase energy, and total solvation energy were −156.21 kcal/mol, −55.97 kcal/mol, −212.18 kcal/mol, and 26.13 kcal/mol, respectively; similarly, in the MMGBSA analysis for vaccine and TLR-3, van der Waals energy, coulombic energy, total gas phase energy, and total solvation energy were −170.66 kcal/mol, −58.17 kcal/mol, −228.83 kcal/mol, and 28.64 kcal/mol, respectively. The docking results showed that the receptor and vaccine have a proper binding affinity. Furthermore, −186.5 kcal/mol and −200.19 kcal/mol net binding energy for vaccine and TLR-2 and vaccine and TLR-3 were predicted, respectively, and the overall energy parameter and calculated energy values are mentioned in **Table 8**.

**Table 8:** Binding free energy estimation and MMGBSA/GBSA analysis.

Parameter	TLR-2	TLR-3
MM-GBSA		
Van der Waals Energy (kcal/mol)	-156.21	-170.66
Columbic Energy (kcal/mol)	-55.97	-58.17
Total Gas Phase Energy (kcal/mol)	- 212.18	- 228.83
Total Solvation Energy (kcal/mol)	26.13	28.64
Net Energy (kcal/mol)	-186.05	-200.19
MM-PBSA		
Van der Waals Energy (kcal/mol)	-156.21	-170.66
Columbic Energy (kcal/mol)	-55.97	-58.17
Total Gas Phase Energy (kcal/mol)	- 212.18	- 228.83
Total Solvation Energy (kcal/mol)	24.02	25.89
Net Energy (kcal/mol)	-188.16	-202.94

### 3.8 Immune simulation analysis

**Figure 9:**
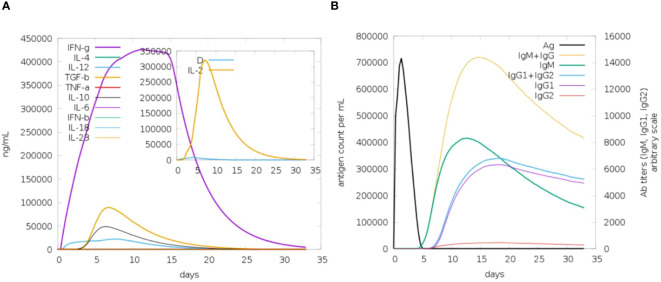
Immune response toward antigenic proteins. **(A)** IFN-g followed by different interleukins (ILs). **(B)** Different antibody responses against the vaccine construct.

The analysis of immune simulation predicted that the construct has properly evoked immune responses in the form of cellular and humoral immune responses; several cytokines were also produced against the vaccine construct, as shown in **Figure 9A**. After cellular immune responses, humoral immune responses were also generated in the form of IgM and IgG, and several other antibodies were also observed as mentioned in **Figure 9B**.

## 4 Discussion

Zika virus infection has been reported from 89 countries as of December 2022 and still no therapeutic drugs and vaccines are available to manage the virus infection. The desired efforts are mainly highlighted for the development of vaccines to eradicate the Zika virus (40). Epitope-based vaccine constructs can properly and safely provoke immune responses on the basis of conserved epitopes in several antigenic proteins (41). The application of an immunoinformatics approach to construct epitope-based vaccine constructs is increasingly common since it can save resources and time during vaccine design and candidate selection against several virus and bacterial species. In protein retrieval, GCA_0008828151, GCA_0023662851, and GCA_0047871951 are mainly involved in the pathogenesis of Zika virus infections. The mentioned proteins were used as potential vaccine candidates and used in epitopes prediction phase. It is well known that the mutation processes in viral species will weaken the effectiveness of the vaccine construct; hence, it is necessary to design a vaccine as effective as possible against the Zika pandemic, as we retrieved and designed a multi-epitope-based vaccine construct against the target pathogen (14, 42). Epitope-based vaccine constructs often create stronger immune response as compared to single-peptide-based vaccines; thus, to enhance the vaccine construct’s immune efficacy, we designed a vaccine construct based on multi-epitopes against the pathogen to provoke immune responses and avoid allergic responses in the host body (6, 43, 44). The epitope-based vaccines comprise non-allergenic, probable antigenic, and non-toxic epitopes (45, 46). Cytokines are signaling molecules and vital for strong immune responses. It was observed that the designed vaccine construct can produce cytokines in high titers, and thus, indicating our vaccine to be a good candidate for vaccine development against the Zika virus (43, 47). The predicted cytokines also help in mediating cellular base immune in order to eradicate the pathogens (48, 49). Receptor molecular docking studies were conducted to analyze the binding interface between designed immune cells and vaccination, as this interaction is crucial for producing an immune response against specific pathogens (21, 50). Molecular docking analysis was used to study the interactions between the vaccine and different immune cells (22, 51). According to the docking study, the vaccine design binds to the target immune cells with appropriate efficiency. Furthermore, docked complexes’ stability is important to provide long-lasting immunity toward the target pathogens (52). MD simulation analysis is a computer-based simulation approach for the analysis of the physical movement of the docked complexes and to assess the dynamic behavior of the docked complexes (25).In molecular dynamics simulation study, it was found that the intermolecular interactions and binding conformation remained stable, and there are high chances that the vaccine could stimulate strong immunological responses (9). The RMSD analysis predicted that the vaccine and the receptor molecules have proper binding ability in a dynamic environment, and it can properly evoke the immune system. Moreover, to confirm the binding interaction of the vaccine construct, a binding free energy calculation analysis was performed following the MMGBSA and MMPBSA analysis approach; in binding free energy, we observed negative binding energy, as a result of binding between vaccine and immune cell receptors, thus representing the proper binding between vaccine constructs and immune cell receptors (25). Overall, the molecular docking analysis and MD simulation analysis reveal that the vaccine and the immune cell receptors have proper stability; hence, the immune system can recognize it easily and generate appropriate immune responses. The C-immune simulation analysis results indicated that the vaccine construct can properly activate both humoral and cellular immune responses; thus, it can be experimentally used while formulating a vaccine against the Zika virus.

## 5 Conclusion

In summary, we have applied robust bioinformatics, immunoinformatics, and different biophysical approaches such as molecular docking and MD simulation analysis in order to design epitope-based vaccine constructs for the Zika virus. For epitope prediction analysis, we selected two proteins, GCA_0008828151 and GCA_0023662851. After epitope prediction, the epitopes were screened through immunoinformatics analysis and prioritized only nine probable antigenic epitopes as potential vaccine candidates. Using the prioritized epitopes, a multi-epitope vaccine construct was designed and subjected for immunoinformatics analysis, which reveals that the vaccine construct has the potential to activate a proper immune response against the Zika virus. Molecular docking and MD simulation analysis predicted that the vaccine and immune cell receptors have a proper binding ability; thus, the vaccine construct can activate the immune response within the host. As we predicted that the vaccine construct can generate immune response against the Zika virus, the construct can also be used in experimental studies; thus, *in vivo* experimental analyses are needed to further confirm the immune efficiency of the vaccine construct.

## Data availability statement

The original contributions presented in the study are included in the article/**Supplementary Material**. Further inquiries can be directed to the corresponding author.

## Author contributions

MA: Writing – original draft. AA: Writing – review & editing. JA: Writing – review & editing. SA: Writing – review & editing. TA: Writing – review & editing. FA: Writing – review & editing. AFA: Writing – review & editing.
