# Supplementary material for: A novel vaccine construct against Zika virus fever: insights from epitope-based vaccine discovery through molecular modeling and immunoinformatics approaches
Source: Front Immunol. 2024 Jul 1;15:1426496. doi: 10.3389/fimmu.2024.1426496 (PMC11267680; doi:10.3389/fimmu.2024.1426496)
Supplement: Supplementary file 1 [file DataSheet_1.docx]

**A Novel Vaccine Construct against Zika virus Fever: Insights from Epitopes based Vaccine Discovery through Molecular Modeling and Immunoinformatics Approaches**

Metab Alharbi^1,*^, Abdulrahman Alshammari^1^, Jawza F. Alsabhan^2^, Sami I. Alzarea^3^, Talal Alshammari^1^, Fawaz Alasmari^1^, Abdullah F. Alasmari^1^

^1^Department of Pharmacology and Toxicology, College of Pharmacy, King Saud University, P.O. Box 2457, Riyadh 11451, Saudi Arabia;

^2^Department of Clinical Pharmacy, college of Pharmacy, King Saud University, Riyadh, Saudi Arabia

^3^Department of Pharmacology, College of Pharmacy, Jouf University, Sakaka, Aljouf 72341, Saudi Arabia

Metab Alharbi [mesalharbi@ksu.edu.sa](mailto:mesalharbi@ksu.edu.sa)

Abdulrahman Alshammari [abdalshammari@ksu.edu.sa](mailto:abdalshammari@ksu.edu.sa)

Jawza F. Alsabhan [Jawza@ksu.edu.sa](mailto:Jawza@ksu.edu.sa)

Sami I. Alzarea [samisz@ju.edu.sa](mailto:samisz@ju.edu.sa)

Talal Alshammari 444105603@student.ksu.edu.sa

Fawaz Alasmari [ffalasmari@KSU.EDU.SA](mailto:ffalasmari@KSU.EDU.SA)

Abdullah F. Alasmari [afalasmari@KSU.EDU.SA](mailto:afalasmari@KSU.EDU.SA)

***Correspondence**: Metab Alharbi [mesalharbi@ksu.edu.sa](mailto:mesalharbi@ksu.edu.sa)

Supplementary Table S1. MHC-II and MHC-I predicted epitopes and their lowest percentile score, predicted by immuno epitopes data base analysis and resources (IEDB).

| MHC-II | Lowest percentile rank | MHC-I | Lowest percentile score |
| --- | --- | --- | --- |
| IRIVNMLKRGVARVN | 0.5 | MLKRGVARV | 0.17 |
|  |  | IRIVNMLKR | 8.4 |
| PLGGLKRLPAGLLLG | 1.7 | GLKRLPAGL | 24 |
|  |  | RLPAGLLLG | 7.1 |
| SLGLINRWGSVGKKE | 0.91 | GLKRLPAGL | 0.24 |
|  |  | PLGGLKRLPA | 26 |
| RSRRAVTLPSHSTRK | 0.6 | VTLPSHSTR | 0.02 |
|  |  | RSRRAVTLP | 0.31 |
| LQTRSQTWLESREYT | 22 | RSQTWLESR | 0.02 |
|  |  | LQTRSQTWL | 2.6 |
| CHHKKGEARRSRRAV | 38 | EARRSRRAV | 0.67 |
|  |  | CHHKKGEAR | 2.3 |
| SDTQYVCKRTLVDRG | 0.17 | TQYVCKRTL | 0.88 |
|  |  | YVCKRTLVDR | 1.6 |
| SRCPTQGEAYLDKQS | 4.2 | RCPTQGEAYL | 0.28 |
|  |  | GEAYLDKQS | 2.7 |
| SDMASDSRCPTQGEA | 5.4 | ASDSRCPTQ | 2.5 |
|  |  | DSRCPTQGEA | 6.6 |
| IPLPWHAGADTGTPH | 2 | IPLPWHAGA | 0.48 |
|  |  | AGADTGTPH | 2.2 |
| HAGADTGTPHWNNKE | 30 | GADTGTPHW | 0.06 |
|  |  | DTGTPHWNNK | 0.43 |
| GPQRLPVPVNELPHG | 0.72 | GPQRLPVPV | 0.18 |
|  |  | VPVNELPHG | 4.5 |
| PVNELPHGWKAWGKS | 3.4 | NELPHGWKAW | 0.01 |
|  |  | HGWKAWGKS | 14 |
| VKNPMWRGPQRLPVP | 26 | NPMWRGPQRL | 0.15 |
|  |  | RGPQRLPVP | 12 |
| AGPLSHHNTREGYRT | 2.5 | LSHHNTREGY | 0.6 |
|  |  | AGPLSHHNTR | 1.1 |
| GYRTQVKGPWHSEEL | 25 | RTQVKGPWH | 0.82 |
|  |  | KGPWHSEEL | 4.1 |
| GPSLRSTTASGRVIE | 0.8 | GPSLRSTTA | 0.2 |
|  |  | TTASGRVIE | 4 |
| GRVIEEWCCRECTMP | 50 | RVIEEWCCR | 0.27 |
|  |  | EWCCRECTMP | 44 |
| ETCGTRGPSLRSTTA | 73 | GPSLRSTTA | 0.2 |
|  |  | ETCGTRGPSL | 0.63 |
| LDPYWGDVKQDLVSY | 2.6 | DVKQDLVSY | 0.01 |
|  |  | LDPYWGDVKQ | 3.6 |
| VSYCGPWKLDAAWDG | 14 | GPWKLDAAW | 0.04 |
|  |  | VSYCGPWKL | 0.76 |
| ALRSGEGRLDPYWGD | 55 | GEGRLDPYW | 0.05 |
|  |  | ALRSGEGRL | 0.8 |
| KNGSYVSAITQGKRE | 0.19 | YVSAITQGK | 0.06 |
|  |  | KNGSYVSAI | 7.6 |
| AITQGKREEETPVEC | 22 | REEETPVEC | 0.41 |
|  |  | AITQGKREE | 19 |
| RWCFDGTTNNTIMED | 1.2 | DGTTNNTIM | 2.4 |
|  |  | RWCFDGTTNN | 9 |
| SVPAEVWTKYGEKRV | 14 | VPAEVWTKY | 0.01 |
|  |  | WTKYGEKRV | 2.8 |
| VVTDIDTMTIDPQVE | 0.08 | DTMTIDPQV | 0.02 |
|  |  | VVTDIDTMTI | 0.65 |
| VVTDIDTMTIDPQVE | 0.08 | DTMTIDPQV | 0.02 |
|  |  | VVTDIDTMTI | 0.65 |
| NPVVDGIVVTDIDTM | 3.5 | NPVVDGIVV | 0.23 |
|  |  | IVVTDIDTM | 0.83 |
| QQRVFKEKVDTRVPD | 0.88 | RVFKEKVDTR | 0.04 |
|  |  | KEKVDTRVPD | 17 |
| EKVDTRVPDPQEGTR | 8.6 | RVPDPQEGTR | 0.66 |
|  |  | EKVDTRVPDP | 42 |
| TTPYGQQRVFKEKVD | 1.8 | TPYGQQRVF | 0.02 |
|  |  | QQRVFKEKVD | 50 |
| LRKPEKVTRWLQSNG |  | KPEKVTRWL | 0.07 |
|  |  | VTRWLQSNG | 3.9 |
| GKVRKDTQEWKPSTG | 6 | KVRKDTQEW | 0.01 |
|  |  | TQEWKPSTG | 12 |
| DTQEWKPSTGWSNWE | 2.8 | QEWKPSTGW | 0.01 |
|  |  | KPSTGWSNWE | 3.7 |
| ELIGRARVSPGAGWS | 3.5 | RARVSPGAGW | 0.1 |
|  |  | ELIGRARVS | 6.6 |
| VPTGRTTWSIHGKGE | 3.4 | RTTWSIHGK | 0.02 |
|  |  | VPTGRTTWSI | 0.21 |
| NDHMEDKTPVTKWTD | 8 | MEDKTPVTKW | 0.01 |
|  |  | NDHMEDKTPV | 17 |
| WCGSLIGHRPRTTWA | 17 | IGHRPRTTW | 0.25 |
|  |  | WCGSLIGHR | 5.35 |
| REDLWCGSLIGHRPR | 19 | REDLWCGSL | 0.12 |
|  |  | GSLIGHRPR | 0.85 |
| KTPVTKWTDIPYLGK | 31 | WTDIPYLGK | 0.69 |
|  |  | KTPVTKWTDI | 0.94 |
| NDHMEDKTPVTKWTD | 0.54 | MEDKTPVTKW | 0.14 |
|  |  | NDHMEDKTPV | 17.65 |
| LWCGSLIGHRPRTTW | 17 | IGHRPRTTW | 0.25 |
|  |  | LWCGSLIGHR | 3.05 |
| VTKWTDIPYLGKRED | 33 | WTDIPYLGK | 0.69 |
|  |  | IPYLGKRED | 3.9 |
| YMDYLSTQVRYLGEE | 3.1 | YMDYLSTQV | 0.3 |
|  |  | TQVRYLGEE | 7.25 |
| GDEEKYMDYLSTQVR | 0.45 | YMDYLSTQV | 0.3 |
|  |  | GDEEKYMDYL | 2.5 |
| RVSPFGGLKRLPAGL | 5.8 | YMDYLSTQV | 0.3 |
|  |  | GDEEKYMDYL | 2.5 |
| SGGFRIVNMLKRGVA | 0.05 | GFRIVNMLK | 0.2 |
|  |  | VNMLKRGVA | 2.3 |
| PKKKSGGFRIVNMLK | 0.09 | GFRIVNMLK | 0.9 |
|  |  | PKKKSGGFR | 14 |
| KPSLGLINRWGSVGK | 17 | KPSLGLINRW | 0.01 |
|  |  | LINRWGSVGK | 0.56 |
| RSRRAVTLPSHSTRK | 1.3 | VTLPSHSTR | 0.02 |
|  |  | RSRRAVTLP | 0.31 |
| LQTRSQTWLESREYT | 0.06 | RSQTWLESR | 0.02 |
|  |  | SQTWLESREY | 0.1 |
| CHHKKGEARRSRRAV | 22 | RSQTWLESR | 0.02 |
|  |  | QTWLESREYT | 4 |
| SDTQYVCKRTLVDRG | 38 | TQYVCKRTL | 0.88 |
|  |  | VCKRTLVDRG | 32 |
| SRCPTQGEAYLDKQS | 0.17 | RCPTQGEAYL | 0.28 |
|  |  | GEAYLDKQS | 2.7 |
| SDMASDSRCPTQGEA | 4.2 | ASDSRCPTQ | 2.5 |
|  |  | DSRCPTQGEA | 6.6 |
| SGMIVNDTGHETDEN | 5.4 | IVNDTGHET | 2.7 |
|  |  | SGMIVNDTGH | 11 |
| GPQRLPVPVNELPHG | 0.41 | GPQRLPVPV | 0.18 |
|  |  | VPVNELPHG | 4.5 |
| VKNPMWRGPQRLPVP | 0.72 | NPMWRGPQRL | 0.15 |
|  |  | RGPQRLPVP | 12 |
| GPSLRSTTASGRVIE | 26 | GPSLRSTTA | 0.2 |
|  |  | TTASGRVIE | 4 |
| GRVIEEWCCRECTMP | 0.8 | RVIEEWCCR | 0.27 |
|  |  | EWCCRECTMP | 72 |
| TCGTRGPSLRSTTAS | 50 | GPSLRSTTA | 0.2 |
|  |  | TCGTRGPSL | 0.84 |
| SNSPIMDTEVEVPER | 74 | DTEVEVPER | 0.52 |
|  |  | SNSPIMDTEV | 1.4 |
| ERAWSSGFDWVTDHS | 0.66 | RAWSSGFDW | 0.03 |
|  |  | SGFDWVTDHS | 18 |
| TRDAFPDSNSPIMDT | 5.3 | FPDSNSPIM | 0.01 |
|  |  | TRDAFPDSNS | 40 |
| QDGLIASLYRPEADK | 3.3 | SLYRPEADK | 0.08 |
|  |  | QDGLIASLY | 0.6 |
| DKVAAIEGEFKLRTE | 0.1 | AAIEGEFKL | 0.14 |
|  |  | EGEFKLRTE | 18 |
| RWCFDGTTNNTIMED | 5.6 | DGTTNNTIM | 2.4 |
|  |  | RWCFDGTTNN | 9 |
| EDSVPAEVWTRHGEK | 1.2 | DSVPAEVWTR | 0.07 |
|  |  | EVWTRHGEK | 0.32 |
| TYTDRRWCFDGTTNN | 8.5 | TYTDRRWCF | 0.04 |
|  |  | RWCFDGTTNN | 9 |
| VVTDIDTMTIDPQVE | 34 | DTMTIDPQV | 0.02 |
|  |  | VVTDIDTMTI | 0.65 |
| NPVVDGIVVTDIDTM | 0.08 | NPVVDGIVV | 0.23 |
|  |  | IVVTDIDTM | 0.83 |
| ALEFYSYKKSGITEV | 3.5 | ALEFYSYKK | 0.52 |
|  |  | YKKSGITEV | 1.2 |
| YSYKKSGITEVCREE | 1.5 | KSGITEVCR | 1.1 |
|  |  | YSYKKSGIT | 6.2 |
| RRALKDGVATGGHAV | 12 | GVATGGHAV | 0.41 |
|  |  | RRALKDGVA | 17 |
| PVKYEEDVNLGSGTR | 3.3 | DVNLGSGTR | 0.21 |
|  |  | PVKYEEDVNL | 9.1 |
| AVVSCAEAPNMKIIG | 0.01 | AEAPNMKII | 0.06 |
|  |  | AVVSCAEAP | 15 |
| QQRVFKEKVDTRVPD | 28 | RVFKEKVDTR | 0.04 |
|  |  | KEKVDTRVPD | 17 |
| TTPYGQQRVFKEKVD | 0.88 | TPYGQQRVF | 0.02 |
|  |  | QQRVFKEKVD | 50 |
| RFWALVDKEREHHLR | 1.8 | RFWALVDKER | 0.22 |
|  |  | DKEREHHLR | 2.6 |
| MGKREKKQGEFGKAK | 3.9 | RFWALVDKER | 0.22 |
|  |  | VDKEREHHL | 0.4 |
| MQDLWLLRRSEKVTN | 27 | MQDLWLLRR | 0.8 |
|  |  | LRRSEKVTN | 27 |
| EKVTNWLQSNGWDRL | 7.9 | VTNWLQSNGW | 0.09 |
|  |  | LQSNGWDRL | 2 |
| GKVRKDTQEWKPSTG | 13 | KVRKDTQEW | 0.01 |
|  |  | TQEWKPSTG | 12 |
| GWDNWEEVPFCSHHF | 2.8 | EEVPFCSHHF | 0.02 |
|  |  | GWDNWEEVPF | 1.6 |
| GRARVSPGAGWSIRE | 88 | RARVSPGAGW | 0.1 |
|  |  | SPGAGWSIRE | 7.5 |
| VPTGRTTWSIHGKGE | 5.2 | RTTWSIHGK | 0.02 |
|  |  | VPTGRTTWSI | 0.21 |
| KTPVTKWTDIPYLGK | 8 | VTKWTDIPY | 0.31 |
|  |  | KTPVTKWTDI | 2.6 |
| REDLWCGSLIGHRPR | 0.54 | GSLIGHRPR | 0.19 |
|  |  | REDLWCGSL | 0.2 |
| NDHMEDKTPVTKWTD | 31 | MEDKTPVTKW | 0.01 |
|  |  | NDHMEDKTPV | 17 |
| GDEEKYMDYLSTQVR | 17 | YMDYLSTQV | 0.03 |
|  |  | GDEEKYMDY | 1 |
| GDEEKYMDYLSTQVR | 5.8 | YMDYLSTQV | 0.03 |
|  |  | GDEEKYMDY | 1 |

Supplementary Table S2. Chi3, Energy and Sum B-Factors obtained from the designed 2.0 online web server.

| Res1 Seq # | Res1 AA | Res2 Seq # | Res2 AA | Chi3 | Energy | Sum B-Factors |
| --- | --- | --- | --- | --- | --- | --- |
| 54 | VAL | 62 | GLU | 102.46 | 4.94 | 0 |
| 68 | GLU | 72 | ASP | 114.98 | 1.25 | 0 |
| 82 | ARG | 89 | GLY | -67.69 | 5.08 | 0 |
| 94 | LYS | 100 | ALA | -57.86 | 3.5 | 0 |
| 120 | LEU | 125 | ALA | 83.15 | 7.75 | 0 |
| 169 | ALA | 185 | ASN | 110.93 | 5.41 | 0 |
| 170 | ARG | 175 | GLY | -104.28 | 6.76 | 0 |
| 174 | PRO | 178 | LYS | -72.19 | 7.52 | 0 |
| 176 | PRO | 184 | ILE | -113.66 | 8.62 | 0 |
| 191 | GLY | 197 | ASP | 74.84 | 5.75 | 0 |
| 203 | GLY | 206 | THR | -82.78 | 5.01 | 0 |
| 215 | GLY | 227 | GLU | 121.96 | 3.73 | 0 |
| 227 | GLU | 232 | PRO | -102.44 | 3.7 | 0 |
| 233 | GLY | 251 | LEU | -100.92 | 5.07 | 0 |
| 236 | ASP | 250 | ASP | 103.64 | 4.1 | 0 |
| 236 | ASP | 251 | LEU | 120.59 | 3.24 | 0 |
| 249 | GLU | 252 | TRP | 105.74 | 5.09 | 0 |
| 252 | TRP | 275 | GLY | 67.3 | 7.13 | 0 |
| 253 | CYS | 263 | ASP | 109.1 | 2.76 | 0 |
| 253 | CYS | 275 | GLY | 95.3 | 3.16 | 0 |
| 261 | GLY | 265 | ALA | 85.27 | 5.55 | 0 |
| 266 | SER | 279 | GLY | -84.9 | 3.26 | 0 |
| 267 | ASP | 270 | CYS | 124.96 | 4.26 | 0 |
| 268 | SER | 282 | LEU | 113.32 | 3.48 | 0 |
| 268 | SER | 292 | ALA | -72.02 | 4.93 | 0 |
| 270 | CYS | 276 | GLY | 123.68 | 5.78 | 0 |
| 292 | ALA | 299 | GLY | 75.1 | 5.91 | 0 |
| 293 | PHE | 298 | SER | 95.24 | 5.73 | 0 |


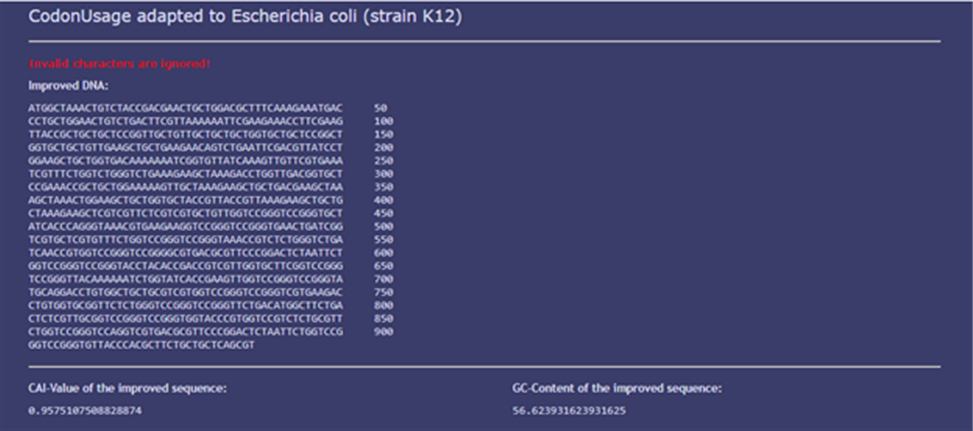


Supplementary Figure S1. Improved DNA sequence, CAI value and GC content of the improved DNA sequence
